# Weight-based stereotype threat in the workplace: consequences for employees with overweight or obesity

**DOI:** 10.1038/s41366-021-01052-5

**Published:** 2021-12-20

**Authors:** Hannes Zacher, Courtney von Hippel

**Affiliations:** 1 Wilhelm Wundt Institute of Psychology, Leipzig University, Leipzig, Germany; 2 School of Psychology, The University of Queensland, Brisbane, QLD Australia

**Keywords:** Obesity, Patient education

## Abstract

**Background/Objectives:**

Employees with overweight or obesity are often stereotyped as lazy, unmotivated, and less competent than employees with normal weight. As a consequence, employees with overweight or obesity are susceptible to stereotype threat, or the concern about confirming, or being reduced to, a stereotype about their group. This survey study examined whether employees with overweight or obesity experience stereotype threat in the workplace, whether it is associated with their perceived ability to meet their work demands (i.e., work ability), and whether high levels of knowledge about one’s self (i.e., authentic self-awareness) can offset a potential negative association.

**Subjects/Methods:**

Using a correlational study design, survey data were collected from *N* = 758 full-time employees at three measurement points across 3 months. Employees’ average body mass index (BMI) was 26.36 kg/m² (SD = 5.45); 34% of participants were employees with overweight (BMI between 25 and <30), and 18% of participants were employees with obesity (BMI > 30).

**Results:**

Employees with higher weight and higher BMI reported more weight-based stereotype threat (*r*s between 0.17 and 0.19, *p* < 0.001). Employees who experienced higher levels of weight-based stereotype threat reported lower work ability, while controlling for weight, height, and subjective weight (*β* = −0.27, *p* < 0.001). Authentic self-awareness moderated the relationship between weight-based stereotype threat and work ability (*β* = 0.14, *p* < 0.001), such that the relationship between stereotype threat and work ability was negative among employees with low authentic self-awareness (*β* = −0.25, *p* < 0.001), and non-significant among employees with high authentic self-awareness (*β* = 0.08, *p* = 0.315).

**Conclusions:**

The findings of this study contribute to the literature by showing that weight-based stereotype threat is negatively associated with employees’ perceived ability to meet their work demands, particularly among those employees with low authentic self-awareness.

People with overweight or obesity face prejudice and discrimination in various aspects of their lives, such as healthcare, education, and interpersonal relationships [1]. Unfortunately, the employment context is no exception. Employees with overweight or obesity are stereotyped to lack self-discipline, self-control, and willpower [2, 3], and are seen as less competent and conscientious [4]. In light of these stereotypes, it is not surprising that cross-sectional surveys, population-based research, and experimental studies demonstrate that people with overweight or obesity experience bias with regard to a variety of workplace outcomes [1]. For example, compared to people with normal weight, people with overweight or obesity are less likely to be hired [5, 6], receive lower pay [7], and are less likely to receive promotions [8]. Employees with overweight or obesity also report being the subject of derogatory comments and other uncivil behaviors from their supervisors and co-workers [9]. In short, employees with overweight or obesity are stigmatized and discriminated against in the workplace [1], raising the clear possibility that these employees will be susceptible to stereotype threat.

According to stereotype threat theory [10], concerns about being stereotyped based on one’s group membership can lead people to psychologically distance themselves from domain-relevant activities and performance. That is, stereotype threat can lead to disidentification—or disengagement—from the task domain. Although the majority of stereotype threat research has taken place in a laboratory setting [11], a growing body of research demonstrates that stereotype threat is also important in the workplace [12, 13]. Research in organizational contexts demonstrates that stigmatized groups (e.g., older employees; women in male-dominated fields) disengage from work when they experience stereotype threat [12, 14]. Given the growing percentage of people with overweight or obesity worldwide [15], it is important to examine whether employees with overweight or obesity experience stereotype threat in the workplace, whether it is associated with their perceived ability to meet their work demands, and whether other psychological factors can offset a potential negative association.

Although the stereotypes about employees with overweight or obesity are varied, many focus on the notion that they are less capable [4]. Over time, people from stereotyped groups can internalize the stigma about their group [16]. If employees with overweight or obesity internalize the stigma that they are less capable at work than their colleagues with normal weight, then feelings of stereotype threat should lead to lowered perceptions of work ability, or the perceived capacity to continue working in their current job given their perceptions of their physical, cognitive, and interpersonal job demands and their ability to meet these demands [17, 18]. In short, employees’ experience of weight-based stereotype threat should lead them to believe they are less capable of meeting the demands of their job.

## The moderating role of authentic self-awareness

Scholarly interest in employee authenticity, or “being your true self at work,” has rapidly increased over the past few years [19]. Authentic self-awareness is the extent of knowledge (and trust in that knowledge) about various aspects of one’s self and the motivation to expand that knowledge [20]. Employees with high levels of authentic self-awareness consider their self as a whole (e.g., physical appearance, internal states including cognitions and emotions, motives and intentions, social commitments) and are invested in understanding and learning more about their “true self” [21]. Research has shown that employees’ authentic self-awareness is empirically distinct from, but moderately and positively associated with self-insight (i.e., clarity of understanding various aspects of one’s self), self-acceptance (i.e., positive attitude to one’s self), and self-esteem (i.e., confidence in one’s own worth), and negatively associated with anxiety, cognitive and emotional strain, and ill-health [21].

We predict that weight-based stereotype threat generally is negatively related to work ability, but that high (vs. low) levels of authentic self-awareness may buffer this negative association. This prediction is based on theorizing that employees who better understand themselves are less likely to comply with unwanted social and situational pressures in their work environment and react less strongly to others’ demands and workplace stressors [21]. Thus, authentic self-awareness may constitute a coping mechanism that helps employees deal with the stressor of weight-based stereotype threat [22, 23]. Consistent with this possibility, experimental work has shown that people who have a more stable sense of self—a trait that is associated with greater self-concept clarity [24] and greater integration of positive and negative information [25]—are more likely to treat negative feedback as a challenge rather than a hindrance [26]. Clarity and stability of the self-concept, as well as integration of positive and negative information into the self-concept, are all important components of authentic self-awareness [20, 27].

Additionally, employees who possess a holistic and differentiated understanding of their self and who are motivated to continuously improve their self-understanding should have a broader and more effective set of psychological coping strategies (e.g., positive reframing, reappraisal) at their disposal when they feel stereotyped [28, 29]. That is, when faced with weight-based stereotype threat, they should be more capable of restoring a sense of themselves as capable employees through consideration of numerous other aspects of their self-concept. In contrast, employees with low authentic self-awareness do not adopt a broad perspective on their self and are less interested in learning more about its elements. With less self-knowledge at their disposal, these employees should be more susceptible to the negative effects of weight-based stereotype threat. Consistent with this possibility, inauthenticity has been hypothesized to result in employees who are more likely to comply with stereotypes [30]. Thus, employees’ authentic self-awareness should moderate the negative relationship between weight-based stereotype threat and work ability, such that weight-based stereotype threat is associated with greater deficits in work ability when authentic self-awareness is low than when authentic self-awareness is high.

## Method

### Participants and procedure

We conducted a correlational survey study with three measurement points over a period of 3 months, incorporating an initial survey with demographic and control variables (Time [T] 1) as well as two subsequent surveys (T2 and T3) that included measures of weight-based stereotype threat, work ability, and authentic self-awareness. We used a time lag of four weeks between measurement points in an effort to ensure that participants could recall their concerns and experiences at work. Data for this study were collected as part of a larger data collection effort, and so far two other studies based on the same dataset, but with completely different research questions and completely different substantive variables, have been published [31, 32]. In Germany, correlational studies are exempt from institutional review board approval. The research was conducted in line with the ethical guidelines and requirements of the German Psychological Society. Participation in the study was voluntary and anonymous, and informed consent was obtained from all participants.

We commissioned a professional and ISO 26362 certified panel provider to recruit participants from a nationally representative online panel in Germany. To be eligible for inclusion, participants had to be at least 18 years old and working full-time. Approximately 3500 participants were initially contacted with a request to participate in the first measurement wave (T1). This number of initial participants was determined based on the panel provider’s recommendations to obtain a final sample size of 750 participants or more at T3, which is sufficient to detect small correlational effect sizes (i.e., *r* ≥ 0.10) with high (i.e., ≥0.80) statistical power [33]. Of those 3500 contacted, 1522 responded and were eligible to participate according to our selected inclusion criteria. Of these 1522 who qualified, 758 consented to participate and provided complete data on all three measurement occasions.

The sample was comprised of 438 (57.8%) men and 320 women (42.2%). Participants’ age ranged from 21 to 74 years with a mean age of 43.83 years (SD = 10.70). Most participants held either a lower-secondary school degree (228; 30.1%), a higher-secondary school degree (137; 18.1%), or a college/university degree (241; 31.8%). Participants worked across 21 different industries, with the public administrative sector (12.7%), manufacturing (12.8%), and healthcare (10.3%) most represented.

Overall, body mass index (BMI) values of participants at T1 ranged from 16.71 to 60.22, with an average BMI of 26.36 kg/m² (SD = 5.45). More specifically, only 17 participants (2%) were employees with underweight (BMI < 18.5), whereas 346 participants (46%) were employees with normal weight (BMI between 18.5 to <25), 260 participants (34%) were employees with overweight (BMI between 25 and <30), and 135 participants (18%) were employees with obesity (BMI > 30).

### Measures

#### Weight-based stereotype threat

We assessed weight-based stereotype threat by self-report at T2 and T3 using an adapted version of a 5-item stereotype threat scale [34, 35], which was itself adapted from a scale to measure stereotype threat in a laboratory context [36]. We adapted the items by referring to participants’ weight instead of their gender or age as in previous studies. Participants were asked to report on their feelings of weight-based stereotype threat within the last 4 weeks. The items followed the introductory statement “Last month (in the last 4 weeks) I worried that…,” and were: “…some people at my workplace felt I have less ability because of my weight,” “…people at my workplace drew conclusions about my ability based on my weight,” “…some people at my workplace felt that I’m not committed to my work because of my weight,” “…some people at my workplace felt that I have less to contribute at work because of my weight,” “…my behavior caused people in my workplace to think that stereotypes about people of my weight are true.” Responses were provided on a five-point scale from 1 (never) to 5 (always). Reliability for the scale was high at both T2 (Cronbach’s *α* = 0.97) and T3 (*α* = 0.98).

#### Authentic self-awareness

We measured employees’ authentic self-awareness at T2 using a four-item scale [21]. Specifically, we asked participants to think about the last 4 weeks when responding to the following items on a five-point scale ranging from 1 (not true at all) to 5 (completely true): “I understood why I thought about myself as I did,” “For better or worse, I knew who I really was,” “I understood well why I behaved like I did,” and “I felt like I didn’t know myself particularly well” (reverse coded). Alpha for the scale was 0.71.

#### Work ability

We measured employees’ perceived work ability at T2 and T3 using a four-item measure [17], which was based on three items from the work ability index [37] and one additional item on interpersonal demands adapted from the work ability index [38]. Participants were asked, “Please evaluate your ability in the last month (the last 4 weeks) to meet the following demands of your work.” The first three items were, “Thinking about the [physical, mental, interpersonal] demands of your work, how do your rate your ability to meet those demands?” and the fourth item was, “How many points would you give your overall ability to work?” Responses were provided on a scale from 0 (was unable to work at all) to 10 (my work ability was at its lifetime best). Reliability for the scale was *α* = 0.92 at T2 and *α* = 0.92 at T3.

#### Control variables

At T1, we assessed employees’ age (in years), gender (1 = male, 2 = female), highest level of education (1 = some high school to 7 = college/university degree), weight (in kilograms), height (in cms), and subjective weight. We measured subjective weight with a single item: “How would you describe your weight?” Responses were provided on a scale ranging from 1 (severely underweight) to 5 (severely overweight). We did not control for BMI, as it was highly correlated with objective and subjective weight (see Table 1).

**Table 1:** Descriptive statistics and correlations.

Variable	*M*	SD	1	2	3	4	5	6	7	8	9	10	11	12
1. Age (T1)	43.83	10.70	–											
2. Gender^a^ (T1)	1.42	0.49	−0.02	–										
3. Education (T1)	5.20	1.60	−0.18**	−0.02	–									
4. Weight^b^ (T1)	81.15	19.88	0.16**	−0.41**	−0.12**	–								
5. Height^c^ (T1)	175.02	9.65	−0.04	−0.67**	0.09*	0.53**	–							
6. Subjective weight (T1)	3.46	0.72	0.21**	0.02	−0.11**	0.59**	−0.06	–						
7. Body mass index (T1)^d^	26.36	5.45	0.22**	−0.13**	−0.19**	0.88**	0.09*	0.74**	–					
8. Weight-based stereotype threat (T2)	1.56	0.94	−0.23**	−0.08*	−0.01	0.19**	0.07	0.11**	0.19**	(0.97)				
9. Weight-based stereotype threat (T3)	1.53	0.93	−0.24**	−0.07	0.02	0.18**	0.08*	0.10**	0.17**	0.76**	(0.98)			
10. Authentic self-awareness (T2)	3.77	0.76	0.26**	0.07*	−0.01	−0.04	−0.01	−0.02	−0.04	−0.41**	−0.36**	(0.71)		
11. Work ability (T2)	8.23	1.81	0.16**	0.06	0.03	−0.09*	0.03	−0.07	−0.13**	−0.35**	−0.28**	0.48**	(0.92)	
12. Work ability (T3)	8.26	1.82	0.15**	0.07	0.05	−0.07	−0.01	−0.04	−0.08*	−0.31**	−0.27**	0.37**	0.60**	(0.92)

*N* = 758. Reliability estimates (*α*), where available, are shown in parentheses along the diagonal.

*T* time.

*p < 0.05; **p < 0.01.

^a^1 = male, 2 = female.

^b^In kilograms.

^c^In centimeters.

^d^kg/m^2^.

## Results

Table 1 shows the means, standard deviations (SD), and correlations of all study variables. Weight, subjective weight, and BMI were positively related to weight-based stereotype threat, whereas age, authentic self-awareness, and work ability were negatively associated with weight-based stereotype threat. An exploratory analysis revealed that weight and BMI did not have curvilinear relationships with weight-based stereotype threat, suggesting that stereotype threat was generally lower among employees with lower weight and lower BMI and higher among employees with higher weight and higher BMI.

Table 2 reports the results of the regression analyses. A Kolmogorov–Smirnov test indicated that our main outcome variable, T3 work ability, was not normally distributed, *D*(758) = 0.108, *p* < 0.001. However, given our large sample size, the fact that there were more than 75 observations per predictor variable, and the sizeable SD (SD = 1.82; see Table 1), this violation of the normality assumption of regression analysis is not a primary concern for this study [39]. Consistent with expectations, weight-based stereotype threat was negatively related to work ability. As shown in Table 2 (Model 1), T2 weight-based stereotype threat was negatively associated with T3 work ability above and beyond the T1 control variables (*β* = −0.27, *p* < 0.001), suggesting that employees who felt higher levels of stereotype threat subsequently perceived lower work ability. Together, weight-based stereotype threat and the control variables explained 11 percent of the variance in work ability. An additional analysis showed that the interaction between gender and weight-based stereotype threat was not significantly associated with work ability (*β* = −0.03, *p* = 0.346), suggesting that the relationship between stereotype threat and work ability is consistent for men and women. The relationship between weight-based stereotype threat and work ability was similar when considering only employees with overweight (i.e., BMI between 25 and <30; *β* = −0.20, *p* = 0.003), only employees with obesity (i.e., BMI > 30; *β* = −0.31, *p* < 0.001), or both of these groups combined in the analysis (*β* = −0.25, *p* < 0.001).

**Table 2:** Results of regression analyses.

	Model 1 DV: T3 work ability			Model 2 DV: T3 work ability			Model 3 DV: T3 work ability			Model 4 DV: T3 weight-based stereotype threat		
	*B*	SE	*β*	*B*	SE	*β*	*B*	SE	*β*	*B*	SE	*β*
Intercept	8.26	0.06		8.38	0.07		8.32	0.06		1.55	0.02	
T1 Age	0.20	0.07	0.11**	0.10	0.07	0.05	0.06	0.06	0.03	−0.07	0.02	−0.07**
T1 Gender	0.17	0.08	0.10*	0.11	0.08	0.06	0.04	0.07	0.02	0.02	0.03	0.02
T1 Education	0.10	0.07	0.05	0.07	0.06	0.04	0.06	0.05	0.03	0.02	0.02	0.02
T1 Weight	−0.04	0.11	−0.02	−0.07	0.11	−0.04	0.06	0.09	0.03	0.06	0.04	0.07
T1 Height	0.15	0.10	0.09	0.11	0.10	0.06	−0.05	0.09	−0.03	0.00	0.04	0.00
T1 Subjective weight	−0.02	0.09	−0.01	−0.03	0.09	−0.02	−0.05	0.08	−0.03	0.00	0.03	0.00
T2 Weight-based stereotype threat	−0.50	0.07	−0.27**	−0.16	0.08	−0.09*	−0.08	0.07	−0.04	0.66	0.02	0.72**
T2 Authentic self-awareness				0.58	0.07	0.32**	0.17	0.07	0.09*	−0.05	0.03	−0.06*
T2 Weight-based stereotype threat × T2 Authentic self-awareness				0.30	0.08	0.14**	0.15	0.07	0.07*			
T2 Work ability							0.94	0.06	0.52**	0.01	0.03	0.01
T2 Work ability × T2 Authentic self-awareness										−0.03	0.02	−0.04
*ΔR*^2^				0.08**			0.19**					
*R*^2^	0.11			0.19			0.38			0.59		
*F*	13.32**			19.11**			45.48**			107.04**		

*N* = 758.

*DV* dependent variable, *T* time.

*p < 0.05; **p < 0.01.

**Fig. 1:**
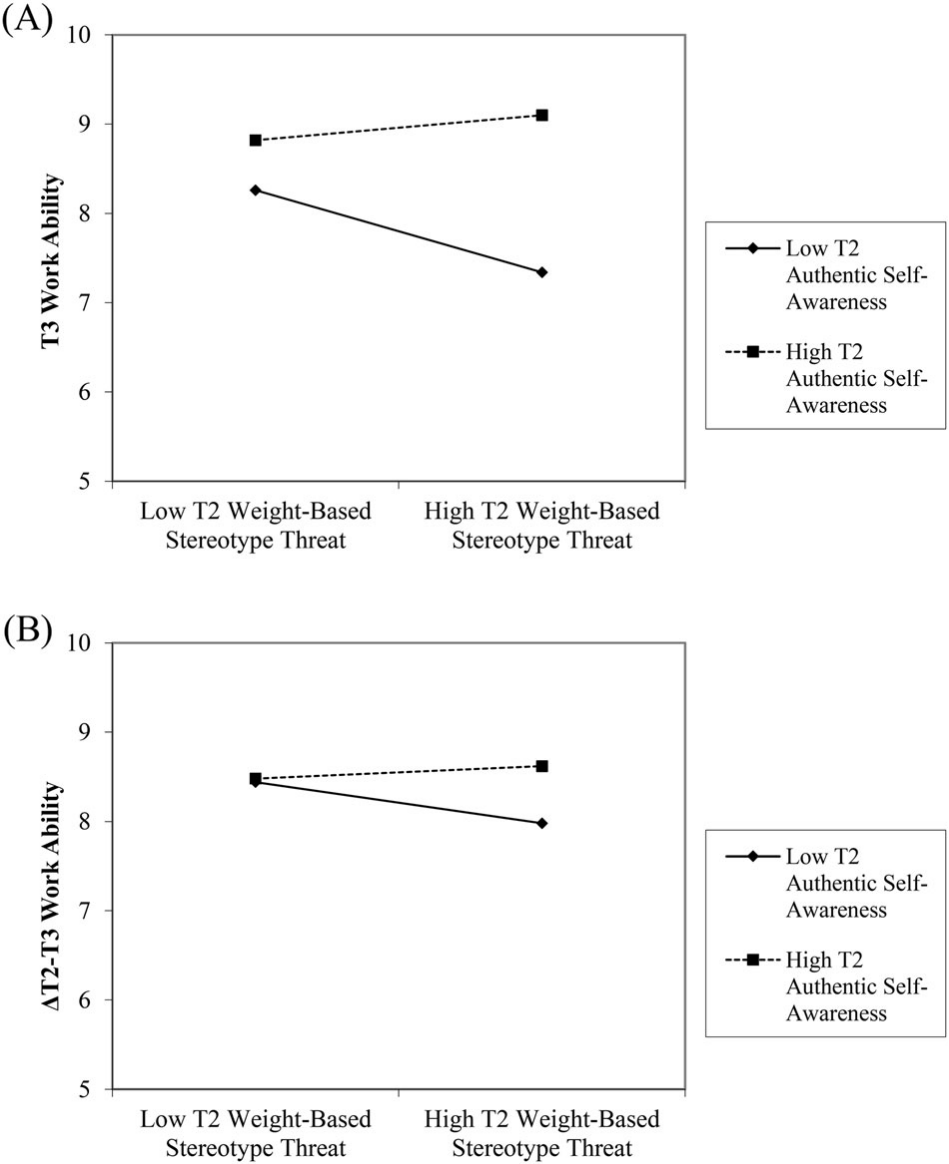
Interaction plots. Effects of T2 weight-based stereotype threat on (**A**) T3 work ability (without controlling for T2 work ability) and (**B**) T3 work ability (controlling for T2 work ability) moderated by T2 authentic self-awareness.

Next, we tested the prediction that authentic self-awareness moderates the relationship between weight-based stereotype threat and work ability. As shown in Table 2 (Model 2), a significant interaction emerged between weight-based stereotype threat and authentic awareness (*β* = 0.14, *p* < 0.001) which, together with the main effect of authentic self-awareness, explained an additional eight percent of the variance in work ability. This interaction is graphically shown in Fig. 1A. Simple slope analyses showed that the relationship between weight-based stereotype threat and work ability was negative and significant at low (−1 SD) levels of authentic self-awareness (*B* = −0.46, SE = 0.08, *β* = −0.25, *t* = −5.80, *p* < 0.001) and weak and non-significant at high (+1 SD) levels of authentic self-awareness (*B* = 0.14, SE = 0.14, *β* = 0.08, *t* = 1.01, *p* = 0.315). An additional analysis showed that a three-way interaction between gender, weight-based stereotype threat, and authentic self-awareness (while controlling for the respective main effects and two-way interaction terms) was not associated with work ability (*β* = −0.03, *p* = 0.413). The interaction between weight-based stereotype threat and authentic self-awareness was also significant when only considering employees with overweight in the analysis (i.e., BMI between 25 and <30; *β* = 0.15, *p* = 0.034), whereas it was not significant when considering only employees with obesity (i.e., BMI > 30; *β* = −0.02, *p* = 0.843) or both of these groups combined in the analysis (*β* = 0.07, *p* = 0.163). Overall, these findings suggest that weight-based stereotype threat was negatively associated with work ability among employees with obesity, as well as among those employees with normal weight or overweight who had low levels of authentic self-awareness. In contrast, weight-based stereotype threat was not significantly associated with work ability among employees with normal weight or overweight who had high levels of authentic self-awareness.

### Supplemental analyses

We conducted a supplemental analysis in which we additionally controlled for baseline (T2) work ability (Model 3, Table 2). The patterns of results of this lagged endogenous change model [40, 41] did not differ substantially from the results reported above. In particular, results suggest that the interaction between weight-based stereotype threat and authentic self-awareness was associated with change in work ability from T2 to T3 (*β* = 0.07, *p* = 0.039). The significant interaction effect is shown in Fig. 1B. Simple slope analyses showed that the relationship between weight-based stereotype threat and change in work ability was negative and significant at low (−1 SD) levels of authentic self-awareness (*B* = −0.23, SE = 0.07, *β* = −0.13, *t* = −3.22, *p* = 0.001) and weak and non-significant at high (+1 SD) levels of authentic self-awareness (*B* = 0.07, SE = 0.12, *β* = 0.04, *t* = 0.55, *p* = 0.585). Again, a three-way interaction between gender, weight-based stereotype threat, and authentic self-awareness was not significantly associated with work ability (*β* = 0.02, *p* = 0.659). Additional analyses with only employees with overweight (*β* = 0.05, *p* = 0.378), only employees with obesity (*β* = −0.03, *p* = 0.729), or both of these groups combined (*β* = 0.01, *p* = 0.852) did not yield significant interaction effects. However, T2 weight-based stereotype threat still had a negative main effect when only considering employees with obesity in this analysis (*β* = −0.20, *p* = 0.026).

To test whether the temporal order of variables we proposed represents the best fit to the data, we also estimated a reverse temporal order model in which we regressed T3 weight-based stereotype threat on the control variables, baseline (T2) weight-based stereotype threat, work ability, authentic self-awareness, and the interaction of work ability and authentic self-awareness (Model 4, Table 2). As shown in Model 4 (Table 2), age (*β* = −0.07, *p* = 0.004) and authentic self-awareness (*β* = −0.06, *p* = 0.044) were weakly and negatively associated with change in weight-based stereotype threat. In contrast, T2 work ability and the interaction between work ability and authentic self-awareness were not significantly associated with change in weight-based stereotype threat. An additional analysis showed that a three-way interaction between gender, work ability, and authentic self-awareness was not significantly associated with change in weight-based stereotype threat (*β* = −0.04, *p* = 0.154). The interaction between work ability and authentic self-awareness was also non-significant when considering only employees with overweight (*β* = 0.06, *p* = 0.092), only employees with obesity (*β* = −0.08, *p* = 0.293), or both of these groups combined (*β* = 0.01, *p* = 0.659).

## Discussion

Consistent with expectations, our correlational survey study showed that the experience of weight-based stereotype threat was associated with lower levels of work ability, and this relationship was qualified by authentic self-awareness. Specifically, the relationship between weight-based stereotype threat and work ability was non-significant among employees with higher authentic self-awareness, whereas employees with lower authentic self-awareness reported lower work ability when they experienced weight-based stereotype threat. This interaction effect was weaker, but still significant, when baseline levels of work ability were controlled, suggesting that the interaction between weight-based stereotype threat and authentic self-awareness is associated with mean-level changes in work ability across 1 month.

These findings, albeit correlational and not causal, advance research on stereotype threat, work ability, and authenticity. Most research on stereotype threat has been conducted in the laboratory, and stereotype threat research in a work context has neglected weight-based stereotype threat [12]. The current results demonstrate that employees with overweight or obesity experience weight-based stereotype threat and this concern, in turn, can diminish their sense of work ability. Work ability is associated with increased absenteeism, disability leave, and early retirement [17], suggesting that weight-based stereotype threat might be indirectly related to these outcomes. Indeed, this has been shown for other forms of stereotype threat in the workplace [42]. Additionally, if stereotype threat is negatively associated with work ability it might create a vicious cycle whereby diminished work ability fuels the stereotypes about people with overweight or obesity, thereby making these employees even more susceptible to stereotype threat.

Our findings are consistent with prior research suggesting that high authentic self-awareness constitutes a psychological resource and coping mechanism [19–21], as it seems to make employees less susceptible to the detrimental consequences of weight-based stereotype threat. It is important to note, however, that authentic self-awareness did not moderate the association between weight-based stereotype threat and work ability when only employees with obesity were considered in the analysis. Due to the fact that these employees are the most overweight and hence the most easily identified as such, it is not surprising that they were also found to be more susceptible to weight-based stereotype threat than employees with overweight or normal weight (i.e., we found positive linear relationships of weight and BMI with weight-based stereotype threat). Thus, it seems likely that authentic self-awareness may only serve a protective function among employees who experience relatively lower levels of stereotype threat (in the current case, employees with overweight, but not those with obesity).

Finally, the bivariate correlations showed that older employees weighed more and had higher BMIs, but nonetheless experienced less weight-based stereotype threat than younger employees. While the former finding is consistent with the literature on work and health [43], a potential explanation for the latter finding is that older employees have accumulated more work and life experience and may, therefore, be less concerned that other people at work reduce them to weight-based stereotypes. Moreover, the positive relationship between age and authentic self-awareness suggests that older employees possess greater knowledge about various aspects of their selves and, thus, may be less susceptible to experiencing weight-based stereotype threat.

### Limitations and future research

This study integrates psychological theorizing on stereotype threat with the literature on obesity and weight stigma to examine the consequences of weight-based stereotype threat in the workplace. Most weight-stigma research focuses on women with overweight [44, 45], whereas our sample includes men and women. Additional analyses showed that two- and three-way interactions of gender with weight-based stereotype threat and authentic self-awareness were not significantly associated with work ability. Nevertheless, examining both genders continues to be important in light of the inconsistent associations of weight stigma with potential outcome variables in a workplace context, with some research showing men to be more susceptible and other research showing women to be more susceptible to weight-based prejudice and discrimination (see [1]). Thus, further research is needed that addresses the issue of intersectionality, or how the combination of gender, weight, and other relevant characteristics may be associated with potential detrimental consequences in the workplace [46, 47].

Nonetheless, this study has a number of limitations that should be addressed in future research. First, all of the constructs in our study were assessed using self-report, which may raise concerns about artificially inflated associations due to common method bias. However, following methodological recommendations [48], we temporally separated measurements of our predictor and outcome variables. Additionally, our predictor, moderator and outcome variables used different response scales, helping to further combat common method variance [49]. Perhaps most importantly, methodologists have demonstrated that interaction effects are not inflated by common method bias [50]. Nonetheless, research demonstrates that self- and other-reports of authentic self-awareness are weak and non-significant [21], suggesting there may be limitations in the accessibility of self-knowledge or that self-presentation biases people’s judgements [51]. To address this possibility, future research could supplement self-report measures of authentic self-awareness with reports obtained from other people (e.g., co-workers, family members).

Second, although work ability is an important outcome, future studies should examine the associations of weight-based stereotype threat with additional engagement- and disengagement-related work outcomes. Research with employees from other stigmatized groups demonstrates that stereotype threat is associated with more negative job attitudes and increased intentions to quit [12]. Lower work ability also relates to more negative attitudes and intentions to quit [17], raising the possibility that work ability may play a mediating role between the experience of stereotype threat and these outcomes.

## Conclusion

Previous research has neglected the potential consequences of weight-based stereotype threat in the work context. Consistent with stereotype threat theory, we found a negative relationship between employees’ experiences of weight-based stereotype threat and work ability, which was weaker among those with higher levels of authentic self-awareness. Thus, organizations should identify ways to enhance authentic self-awareness, particularly among employees who may be susceptible to the negative effects of weight-based stereotype threat.
